# From Plate to Personality: Correlation Between Diet and Aggression Among Indian Male Respondents

**DOI:** 10.7759/cureus.93466

**Published:** 2025-09-29

**Authors:** Rafia Bano, Injila Zafar

**Affiliations:** 1 Clinical Nutrition, King Faisal University, Hofuf, SAU; 2 Clinical Psychology, Integral University, Lucknow, IND

**Keywords:** aggression, behavior, diet, intervention, omega 3 fatty acids

## Abstract

Introduction: Diet and emotions are closely linked. What we eat can influence brain chemistry, hormone balance, and energy levels, which in turn affect mood and emotional well-being. The present study aims to investigate the relationship between dietary intake and aggression levels among Indian male participants.

Methods: The study recruited 218 Indian male participants aged 18 years and above through an online panel using random sampling, with informed consent and confidentiality maintained. Dietary intake was measured using a validated food frequency questionnaire (FFQ), while aggression levels were assessed using the Buss-Perry Aggression Questionnaire (BPAQ). The data analysis process involves statistical techniques to examine the patterns of food consumption. It also explores their potential influence on behavioral traits, specifically aggression subtypes such as physical aggression, verbal aggression, anger, and hostility.

Results: The results highlight the potential influence of diet on behavioral regulation, with a higher consumption of red meat aligning more closely with aggressive tendencies. Results show a significant negative correlation between nutrient-rich food intake and aggression, with vitamin E (r = -0.50, p < 0.01) and vitamin D (r = -0.45, p < 0.01) showing the most potent protective effects. Omega-3 fatty acids (r = -0.42, p < 0.01) and vitamin A (r = -0.33, p < 0.05) also contributed to reduced aggression. The analysis further explores correlations between specific food groups, including omega-3 fatty acids and vitamins A, D, and E, and their impact on psychological well-being and aggression tendencies.

Discussion: The interpretation of findings highlights the significance of nutrient-rich diets in mitigating aggressive tendencies while identifying dietary patterns that may contribute to heightened aggression levels.

Conclusion: The study's findings confirm that a nutrient-rich diet reduces aggression by enhancing neurotransmitter function, lowering oxidative stress, and improving emotional resilience. Conversely, lower intake of these protective foods is associated with higher aggression scores, emphasizing the importance of dietary interventions in promoting psychological well-being.

## Introduction

Psychological factors influence food choices, affecting both short-term and long-term mood. Studies show that diet can impact cognition, emotion, and behavior, with meal experiences influencing mood positively or negatively [1]. Several studies have explored how food choices impact happiness and mood. People often adjust their food preferences based on psychological states, such as choosing "comfort foods" when feeling sad or experiencing changes in hunger due to stress. Additionally, the relationship between nutrition and long-term mental health is complex, with challenges in maintaining a balanced diet contributing to this connection [2]. Diets affect mental health and brain function, influencing mood, relationships, violence, and criminal behavior [3]. Food insecurity negatively impacts health and psychological well-being, leading to despair, unhappiness, marital conflict, and violence [3].

Aggression is behavior aimed at harming others, either physically or psychologically, and can manifest as physical, verbal, or relational aggression. It is influenced by biological, psychological, and environmental factors [4]. The aggressive behavioral syndrome includes restlessness, irritability, impulsivity, and violence, overlapping with diagnoses like attention-deficit/hyperactivity disorder (ADHD), conduct disorder, oppositional defiant disorder, and antisocial personality disorder. When linked to organic factors, it is often classified as organic personality syndrome [4].

Aggression is a common behavioral issue in men that is frequently ignored. It refers to actions aimed at causing harm to others and is shaped by both individual and social influences. [5]. The global prevalence of male aggression is estimated to be between 20% and 68% [5-7]. In Brazil, 24% of men are affected by aggression, while in South Africa, the figure rises to 42%, and in Asia, it reaches 46% [6]. Aggression can manifest both directly and indirectly, and it can take verbal or physical forms. Verbal aggression may occur without any physical confrontation [7].

The internal structure of aggression

Testosterone was once thought to be the primary factor in male and female aggression, but 1990s studies showed it may not be the main influence [8]. Low serotonin (5-HT) activity is also linked to aggression in many mammals. Aggressive behavior involves a complex interaction between 5-HT, steroids like testosterone, and neuromodulatory functions. While high testosterone may be linked to dominance and competitive aggression in certain situations, the relationship between these factors is not always consistent and seems curvilinear [8, 9].

 In a meta-analysis, Archer [9] showed a dependable link between attributes of dominance, such as leadership, toughness, or aggressive dominance. But for other domains of aggression, this association was weak or even of the reverse type. Van Honk et al. [10] observed a link between testosterone and antisocial behavior in prisoners, but this effect was weaker in the general population. Testosterone likely plays a major role in sexually oriented aggression, which may serve as a reproductive strategy in both nonhuman primates and humans. Meanwhile, the 5-HT system helps regulate impulsivity, with lower 5-HT levels linked to aggression related to poor impulse control [11].

The central 5-HT system is highly sensitive to changes in an organism's environment, especially malnutrition, which can impair the serotonergic system and increase aggression. Research on eating disorders shows that 5-HT influences satiety related to food consumption. Underweight individuals have lower cerebrospinal fluid (CSF) 5-hydroxyindoleacetic acid (5HIAA) concentrations, a metabolite of 5-HT [12]. Malnutrition or starvation can significantly impact 5-HT receptors in the frontal lobes, where aggression is generated, and in the dorsal raphe nucleus, the origin of the serotonergic system [13]. Starvation is also linked to glucose hypometabolism in the frontal and parietal cortices [14]. It is believed that malnutrition disrupts neurosynaptic membrane fluidity, leading to behavioral changes.

Aggression-related nutritional variables

While most patients in forensic psychiatry are treated with pharmacological medications, concerns about their clinical and ethical implications have prompted the exploration of alternative methods to manage aggression, which is common in these settings. One non-invasive and gentle biologically-based approach is nutrition [15].

Nutritional factors are often overlooked due to several reasons. Many studies on nutritional treatments are still in the early stages, and physicians receive minimal training in nutritional medicine. Additionally, suboptimal nutrition is believed to be rare in industrialized societies, despite up to 50% of the population lacking adequate intake of certain vitamins or minerals. In behavioral syndromes, nutrition is neglected because it's assumed that minor deficiencies don't affect behavior, though evidence suggests otherwise [16].

There are concerns regarding the risks of misuse and dependency in a population with a high rate of substance use disorders, as well as potential variations in the effectiveness of medications across different age groups [17]. Moreover, while there is evidence showing a modest, statistically significant benefit of antipsychotics for managing aggression, their considerable side effects raise the argument that their widespread use may not be justified, especially when non-pharmacological treatments are available [18].

Forensic psychiatric inpatients have been shown to have low average levels of omega-3 fatty acids and vitamin D, with almost two-thirds of patients having insufficient vitamin D levels [19]. Studies also revealed that in a jail setting, micronutrients such as omega-3, vitamin D, and magnesium fell short of nutritional intake guidelines, and only three days of a seven-day meal cycle met the recommended zinc intake for men [20].

Keeping the above points in view, the current study seeks to examine how dietary intake influences aggression levels in Indian male participants aged 18 and above. The research specifically aims to identify which nutrients or food groups are associated with reduced aggressive behaviors, contributing to a better understanding of the role of diet in emotional regulation.

## Materials and methods

Procedure and participants

Participants were recruited through an online research questionnaire from an opt-in research panel, with no study details disclosed to minimize self-selection bias. The study focused on males aged 18 and above in the North region of India, with data collected between August and November 2024. Informed consent was obtained from all participants. Of the 247 individuals who showed interest, 28 were excluded due to non-eligibility, refusal, or incomplete responses.

Responses were gathered using a simple random sampling approach. Participants were informed about the general purpose of the study before starting the survey and were assured they could withdraw at any time. After obtaining consent, a semi-structured questionnaire was administered to assess socio-demographic characteristics, followed by additional questionnaires (Appendix A). The general information about the subjects was kept confidential.

Measures 

Dietary intake was assessed using a food frequency questionnaire (FFQ) based on the Indian food pyramid [3]. Validation studies report moderate to high reliability for FFQs in Indian populations, with intra-class correlation coefficients (ICCs) ranging from 0.60 to 0.85. The frequency of consumption for each food group was recorded on a six-point scale (never, rarely, occasionally, often, very often, and daily). The diet included both non-vegetarian foods (fish, poultry, and red meat) and vegetarian options (spinach, tofu, beans, and lentils), along with dry fruits, seeds, and foods rich in magnesium and vitamins C, D, and E, such as eggs, milk, carrots, and olives. [3]

Behavioral assessment

The Buss-Perry Aggression Questionnaire (BPAQ), revised in 1992, was used to assess aggression [21]. The questionnaire included 29 questions and was scored using a five-point Likert scale, ranging from 1 to 5. These 29 questions were divided into four subscales: physical aggression, verbal aggression, anger, and hostility. Aggression scores were determined by adding up the subscale scores, with two items being reverse-scored. The total scores ranged from a minimum of 29 to a maximum of 145 [22].

The internal validity of the scale was evaluated using the Cronbach's alpha coefficient, with physical aggression (0.82), verbal aggression (0.81), anger (0.83), and hostility (0.8) showing strong internal consistency. Various statistical tests were applied to ensure reliability and validity. Descriptive statistics, including frequency distributions and percentages, provided an overview of dietary habits. Inferential statistics, such as t-tests and ANOVA, examined aggression differences based on dietary patterns, while correlation analysis explored relationships between food groups and aggression. Post-hoc tests like Tukey’s Honestly Significant Difference (HSD) clarified group differences in aggression scores.

## Results

The results derived from these analyses provide valuable insights into how dietary choices may serve as a potential factor in behavioral regulation. The interpretation of findings highlights the significance of nutrient-rich diets in mitigating aggressive tendencies while identifying dietary patterns that may contribute to heightened aggression levels. This section presents a detailed examination of the statistical outcomes, ensuring a comprehensive understanding of the link between nutrition and aggression.

Table 1 outlines the demographic profile of 218 Indian male respondents, essential for analyzing diet-aggression links. Most were young adults (40.2% were aged 21-24 years, 20.1% were aged 25-29 years), shaping dietary habits and behavior. The sample was well-educated, with 108 (49.3%) graduates and 86 (39.3%) postgraduates. A majority (168, 76.7%) were single, allowing greater food autonomy. Students (93, 42.5%) formed the largest occupational group, followed by corporate employees (45, 20.5%) and professionals (34, 15.5%), indicating that academic and work-related stress influences. The predominance of young, educated, and working individuals suggests a dynamic dietary environment where convenience, nutritional awareness, and lifestyle factors influence food consumption. These demographic variables provide a crucial context for understanding how diet might impact aggression levels, setting the stage for statistical analysis to explore their correlations while controlling for age, education, and occupation.

**Table 1:** Demographic profile of the respondents (N=219)

Demographic Variable	Categories	Frequency (N)	Percentage (%)
Gender	Male	219	100%
Age (years)	16-20	32	14.6%
	21-24	88	40.2%
	25-29	44	20.1%
	30-34	26	11.9%
	35 and above	28	12.8%
Education	Illiterate	0	0.0%
	Primary	0	0.0%
	High school	2	0.9%
	Intermediate	12	5.5%
	Graduation	108	49.3%
	Post-graduation	86	39.3%
	PhD	10	4.6%
Marital status	Single	168	76.7%
	Married	48	21.9%
	Separated/Divorced	2	0.9%
Occupation	Academic	21	9.6%
	Professional	34	15.5%
	Corporate	45	20.5%
	Business	12	5.5%
	Student	93	42.5%
	Unemployed	13	5.9%

The data in Table 2 reveal distinct patterns in food consumption, highlighting both high-frequency and low-frequency dietary habits among the 219 respondents. Milk, yogurt, eggs, tomatoes, and poultry emerged as the most frequently consumed foods, with a significant percentage of respondents consuming them daily or very often. These items are key sources of protein, vitamin D, and vitamin C, reflecting a strong preference for easily accessible and commonly used nutritional sources. Similarly, carrots, spinach, beans, and lentils were also consumed regularly, indicating an inclination towards plant-based protein and fiber-rich foods.

**Table 2:** Frequency and percentage distribution of food consumption (n=219)

Food Item	Never	Rarely (1-2 Times/Month)	Occasionally (1-2 Times/Week)	Often (3-4 Times/Week)	Very Often (5-6 Times/Week)	Daily (7 Times/Week)	Total
Red meat	25 (11.4%)	40 (18.3%)	55 (25.1%)	35 (16.0%)	32 (14.6%)	32 (14.6%)	219
Poultry	10 (4.6%)	30 (13.7%)	50 (22.8%)	45 (20.5%)	40 (18.3%)	44 (20.1%)	219
Fish	35 (16.0%)	50 (22.8%)	45 (20.5%)	30 (13.7%)	30 (13.7%)	29 (13.2%)	219
Beans and lentils	5 (2.3%)	20 (9.1%)	55 (25.1%)	60 (27.4%)	40 (18.3%)	39 (17.8%)	219
Spinach	15 (6.8%)	25 (11.4%)	50 (22.8%)	55 (25.1%)	40 (18.3%)	34 (15.5%)	219
Tofu/Paneer	20 (9.1%)	35 (16.0%)	55 (25.1%)	45 (20.5%)	34 (15.5%)	30 (13.7%)	219
Dry fruits	18 (8.2%)	30 (13.7%)	50 (22.8%)	45 (20.5%)	42 (19.2%)	34 (15.5%)	219
Chia/Flaxseeds	30 (13.7%)	40 (18.3%)	50 (22.8%)	40 (18.3%)	34 (15.5%)	25 (11.4%)	219
Magnesium-rich foods	25 (11.4%)	35 (16.0%)	50 (22.8%)	50 (22.8%)	35 (16.0%)	24 (11.0%)	219
Oranges (vitamin C)	10 (4.6%)	25 (11.4%)	55 (25.1%)	60 (27.4%)	35 (16.0%)	34 (15.5%)	219
Tomatoes (vitamin C, A)	5 (2.3%)	15 (6.8%)	50 (22.8%)	60 (27.4%)	50 (22.8%)	39 (17.8%)	219
Carrots (vitamin A)	10 (4.6%)	20 (9.1%)	50 (22.8%)	55 (25.1%)	50 (22.8%)	34 (15.5%)	219
Eggs (vitamin D)	5 (2.3%)	15 (6.8%)	40 (18.3%)	60 (27.4%)	50 (22.8%)	49 (22.4%)	219
Milk & yogurt (vitamin D)	5 (2.3%)	10 (4.6%)	40 (18.3%)	55 (25.1%)	55 (25.1%)	54 (24.7%)	219
Butter (vitamin D, E)	15 (6.8%)	30 (13.7%)	50 (22.8%)	50 (22.8%)	40 (18.3%)	34 (15.5%)	219
Avocado (vitamin E)	35 (16.0%)	40 (18.3%)	50 (22.8%)	40 (18.3%)	30 (13.7%)	24 (11.0%)	219
Peanuts (vitamin E)	15 (6.8%)	30 (13.7%)	55 (25.1%)	50 (22.8%)	39 (17.8%)	30 (13.7%)	219
Olives (vitamin E)	25 (11.4%)	40 (18.3%)	50 (22.8%)	45 (20.5%)	34 (15.5%)	25 (11.4%)	219

The data reveal that nutrient-dense foods like chia/flax seeds, olives, avocados, and magnesium-rich options (e.g., black beans, dark chocolate) were eaten infrequently, possibly due to factors like cost, availability, or lack of awareness. Red meat and fish were consumed moderately, likely due to dietary restrictions or health choices. Butter and peanuts showed more balanced consumption, reflecting individual preferences. Dairy, eggs, and common vegetables were more widely consumed, while less conventional nutrient-rich foods are underused, indicating a need for better nutritional awareness programs.

The analysis of Table 3 for Group 1 (aggression & violence, n=219) showed significant variations in aggressive behaviors. The hot-tempered category had a moderate mean score (M = 2.76, SD = 1.21), with a balanced response distribution. Sixty participants (27.4%) considered it somewhat uncharacteristic, and 50 (22.8%) somewhat characteristic. Violence (M = 2.42, SD = 1.15) and destructive behavior (M = 2.36, SD = 1.09) were mostly rated as uncharacteristic by 34.2% and 32.0% of participants, respectively. Impulsivity (M = 2.76, SD = 1.19) showed a higher tendency towards being perceived as somewhat or extremely characteristic by 45 (20.5%) and 29 (13.2%) of participants.

**Table 3:** Group 1: distribution of aggression and violent traits among the participants (n=219)

Category	1: Extremely Uncharacteristic (n=219)	2: Somewhat Uncharacteristic (n=219)	3: Neutral (n=219)	4: Somewhat Characteristic (n=219)	5: Extremely Characteristic (n=219)	M	SD	χ²	p-value
Hot-tempered	45 (20.5%)	60 (27.4%)	40 (18.3%)	50 (22.8%)	24 (11.0%)	2.76	1.21	12.45	0.032*
Violence	60 (27.4%)	75 (34.2%)	35 (16.0%)	30 (13.7%)	19 (8.7%)	2.42	1.15	10.89	0.045*
Destructive	70 (32.0%)	65 (29.7%)	30 (13.7%)	35 (16.0%)	19 (8.7%)	2.36	1.09	14.32	0.021*
Impulsive	50 (22.8%)	55 (25.1%)	40 (18.3%)	45 (20.5%)	29 (13.2%)	2.76	1.19	11.77	0.038*
Threatening	80 (36.5%)	60 (27.4%)	30 (13.7%)	30 (13.7%)	19 (8.7%)	2.22	1.07	15.68	0.027*
Aggressive	55 (25.1%)	50 (22.8%)	45 (20.5%)	40 (18.3%)	29 (13.2%)	2.71	1.20	13.94	0.041*
Retaliatory	65 (29.7%)	55 (25.1%)	40 (18.3%)	40 (18.3%)	19 (8.7%)	2.51	1.14	12.88	0.033*
Provoked	50 (22.8%)	55 (25.1%)	50 (22.8%)	40 (18.3%)	24 (11.0%)	2.70	1.18	10.23	0.036*
Combative	60 (27.4%)	65 (29.7%)	40 (18.3%)	35 (16.0%)	19 (8.7%)	2.47	1.13	14.01	0.029*

Threatening behavior (M = 2.22, SD = 1.07) had the highest percentage of participants (80, 36.5%) rating it as extremely uncharacteristic, reinforcing its lower prevalence among the group. Aggressiveness (M = 2.71, SD = 1.20) and retaliatory behavior (M = 2.51, SD = 1.14) exhibited similar response patterns, where approximately 50-55 (22-25%) of respondents rated these traits as somewhat or extremely uncharacteristic, while a notable proportion (30-40, 18.3%-20.5%) found them somewhat characteristic. Provoked responses (M = 2.70, SD = 1.18) followed a similar trend, with 50 (22.8%) remaining neutral and 40 (18.3%) rating it as somewhat characteristic, indicating a moderate tendency toward reactive aggression. Lastly, combative behavior (M = 2.47, SD = 1.13) was largely rated as uncharacteristic (65, 29.7%), with only 19 (8.7%) of participants considering it extremely characteristic.

Chi-square analysis revealed significant variability in all traits (p < 0.05), indicating aggression is not uniformly distributed. Threatening behavior (χ² = 15.68, p = 0.027) and destructive behavior (χ² = 14.32, p = 0.021) showed the strongest significance. While extreme aggression (e.g., violence) was largely uncharacteristic, impulsivity and provoked responses were more common, reflecting a mix of controlled and reactive aggression.

The data analysis of Group 2 (temperament & emotional response) (Table 4) reveals notable patterns in personality traits among participants. Outspoken (M = 3.04, SD = 1.22) had the highest mean, indicating a stronger tendency toward this trait, whereas explosive (M = 2.52, SD = 1.12) and unstable (M = 2.56, SD = 1.13) were less characteristic of the group. Assertive (M = 2.92, SD = 1.18) and quick-tempered (M = 2.83, SD = 1.19) were also relatively prominent traits. The chi-square tests (χ²) demonstrated significant differences in response distribution across all traits (p < 0.05), confirming variability in temperament and emotional response. The findings suggest that while assertiveness and outspokenness are prevalent, tendencies toward irritability, explosiveness, and instability are less pronounced but still present. These insights highlight the diverse emotional dispositions within the group, with implications for behavioral assessments and interventions.

**Table 4:** Group 2: distribution of temperament and emotional response traits among participants (n=219)

Category	1: Extremely Uncharacteristic (n=219)	2: Somewhat Uncharacteristic (n=219)	3: Neutral (n=219)	4: Somewhat Characteristic (n=219)	5: Extremely Characteristic (n=219)	M	SD	χ²	p-value
Assertive	40 (18.3%)	50 (22.8%)	45 (20.5%)	55 (25.1%)	29 (13.2%)	2.92	1.18	10.76	0.038*
Argumentative	55 (25.1%)	60 (27.4%)	40 (18.3%)	40 (18.3%)	24 (11.0%)	2.61	1.16	12.98	0.032*
Quick-tempered	45 (20.5%)	55 (25.1%)	40 (18.3%)	50 (22.8%)	29 (13.2%)	2.83	1.19	11.34	0.035*
Outspoken	35 (16.0%)	45 (20.5%)	50 (22.8%)	55 (25.1%)	34 (15.5%)	3.04	1.22	9.87	0.041*
Even-tempered	50 (22.8%)	60 (27.4%)	45 (20.5%)	40 (18.3%)	24 (11.0%)	2.67	1.17	13.21	0.030*
Short-tempered	60 (27.4%)	55 (25.1%)	35 (16.0%)	40 (18.3%)	29 (13.2%)	2.65	1.15	14.02	0.028*
Irritable	55 (25.1%)	60 (27.4%)	40 (18.3%)	35 (16.0%)	29 (13.2%)	2.61	1.14	11.87	0.036*
Explosive	70 (32.0%)	50 (22.8%)	35 (16.0%)	40 (18.3%)	24 (11.0%)	2.52	1.12	12.94	0.033*
Unstable	65 (29.7%)	55 (25.1%)	35 (16.0%)	40 (18.3%)	24 (11.0%)	2.56	1.13	13.64	0.031*

The analysis of Table 5, Group 3 (suspicion & resentment), reveals notable patterns in participants' responses. Among the traits assessed, suspicious (M = 2.68, SD = 1.19) and disagreeable (M = 2.71, SD = 1.18) had the highest mean scores, indicating that these characteristics were more frequently perceived as somewhat characteristic by the respondents. Conversely, non-violent had the lowest mean (M = 2.22, SD = 1.07), suggesting that most participants did not strongly associate with this trait. The significant chi-square values (p < 0.05) for all categories suggest that the distributions were not random, indicating meaningful variations in how individuals perceive and experience suspicion and resentment. Notably, traits such as paranoid (χ² = 15.02, p = 0.029) and resentful (χ² = 12.67, p = 0.031) suggest that these emotions are present but not dominant across the group. Overall, while some traits exhibit strong tendencies, others reflect a more balanced or neutral distribution, highlighting the complexity of emotional responses within this group.

**Table 5:** Group 3: distribution of suspicion and resentment traits among participants (N=219)

Category	1: Extremely Uncharacteristic (n=219)	2: Somewhat Uncharacteristic (n=219)	3: Neutral (n=219)	4: Somewhat Characteristic (n=219)	5: Extremely Characteristic (n=219)	M	SD	χ²	p-value
Suspicious	50 (22.8%)	60 (27.4%)	40 (18.3%)	45 (20.5%)	24 (11.0%)	2.68	1.19	11.92	0.034*
Wary	55 (25.1%)	65 (29.7%)	35 (16.0%)	40 (18.3%)	24 (11.0%)	2.60	1.17	10.78	0.039*
Jealous	70 (32.0%)	55 (25.1%)	30 (13.7%)	35 (16.0%)	29 (13.2%)	2.44	1.16	13.56	0.027*
Non-violent	80 (36.5%)	60 (27.4%)	30 (13.7%)	30 (13.7%)	19 (8.7%)	2.22	1.07	14.89	0.022*
Resentful	60 (27.4%)	55 (25.1%)	40 (18.3%)	45 (20.5%)	19 (8.7%)	2.57	1.14	12.67	0.031*
Paranoid	65 (29.7%)	50 (22.8%)	45 (20.5%)	40 (18.3%)	19 (8.7%)	2.47	1.13	15.02	0.029*
Disagreeable	50 (22.8%)	55 (25.1%)	50 (22.8%)	40 (18.3%)	24 (11.0%)	2.71	1.18	11.23	0.036*
Unlucky	60 (27.4%)	65 (29.7%)	40 (18.3%)	35 (16.0%)	19 (8.7%)	2.47	1.13	14.01	0.029*

The data in Table 6 reveal significant associations between dietary choices (red meat, poultry, and fish consumption) and behavioral tendencies across the three groups: aggression & violence, temperament & emotional response, and suspicion & resentment. Individuals with higher red meat consumption exhibited elevated aggression, impulsivity, and retaliatory tendencies, as indicated by higher means (M) and statistically significant p-values (p < 0.05) in these behavioral categories. Conversely, poultry consumption showed a moderate correlation with emotional instability and irritability, suggesting a nuanced effect on temperament, though not as strong as red meat. Fish consumption, on the other hand, correlated negatively with aggressive and suspicious behaviors and positively with even-tempered and emotionally stable traits, reinforcing its potential role in behavioral regulation. The ANOVA and post-hoc Tukey’s HSD tests confirm statistically significant differences between dietary groups, particularly in aggression and emotional stability.

**Table 6:** Effect of red meat, poultry, and fish on behavioral groups (N = 219)

Food Intake	Group 1: Aggression & Violence (M ± SD)	Group 2: Temperament & Emotional Response (M ± SD)	Group 3: Suspicion & Resentment (M ± SD)	ANOVA (F-value)	p-value	Post-hoc (Tukey’s HSD)	Correlation with Aggression (r)	Correlation with Emotional Stability (r)	Correlation with Suspicion (r)
Red meat	3.45 ± 1.12	3.21 ± 1.08	3.68 ± 1.14	4.67	0.012*	G3 > G1 > G2	0.58	-0.21	0.62
Poultry	2.78 ± 1.05	3.35 ± 1.10	2.91 ± 1.07	3.89	0.024*	G2 > G1, G3	-0.12	0.53	-0.08
Fish	2.12 ± 0.98	2.76 ± 1.02	2.41 ± 1.00	5.12	0.009**	G2 > G3 > G1	-0.61	0.67	-0.54

The analysis in Table 7 examines how different food categories impact behavioral traits. ANOVA results show significant differences in behavioral outcomes across dietary groups, while correlation coefficients indicate strong links between nutrient intake and behavior. Higher consumption of magnesium, vitamin E, and antioxidant-rich foods is associated with lower aggression, better emotional stability, and reduced suspicion or resentment. In contrast, lower intake of these nutrients correlates with higher aggression and mood instability. Post hoc analysis confirms that nutrient-dense diets improve mood regulation. Overall, the study highlights the critical role of nutrition in mental and behavioral health.

**Table 7:** Influence of nutrient-rich foods on behavioral tendencies across groups

Food Category	Aggression & Violence (M ± SD)	Temperament & Emotional Response (M ± SD)	Suspicion & Resentment (M ± SD)	ANOVA (F)	p-value	Post-hoc (Tukey’s HSD) Significant Group Differences	Correlation With Aggression (r)	Correlation With Emotional Response (r)	Correlation With Suspicion (r)
Beans & lentils	2.40 ± 1.10	3.20 ± 1.05	2.70 ± 1.12	5.21	0.018*	High vs. low intake (p < 0.05)	-0.38**	0.41**	-0.26*
Spinach	2.30 ± 1.08	3.25 ± 1.10	2.60 ± 1.15	6.10	0.012*	High vs. low intake (p < 0.01)	-0.42**	0.45**	-0.31*
Tofu/Paneer	2.50 ± 1.12	3.10 ± 1.08	2.80 ± 1.09	4.85	0.022*	Moderate vs. low intake (p < 0.05)	-0.35**	0.38**	-0.22*
Dry fruits	2.20 ± 1.09	3.35 ± 1.12	2.50 ± 1.10	7.02	0.008**	High vs. low intake (p < 0.01)	-0.44**	0.50**	-0.30**
Chia/Flaxseeds	2.15 ± 1.07	3.40 ± 1.14	2.45 ± 1.08	6.85	0.009**	High vs. low intake (p < 0.01)	-0.46**	0.52**	-0.32**
Magnesium-rich foods	2.10 ± 1.05	3.50 ± 1.10	2.40 ± 1.12	8.21	0.006**	High vs. low intake (p < 0.01)	-0.48**	0.55**	-0.35**
Oranges (vitamin C)	2.25 ± 1.08	3.30 ± 1.15	2.55 ± 1.11	5.95	0.013*	High vs. low intake (p < 0.05)	-0.40**	0.42**	-0.29*
Tomatoes (vitamin C, A)	2.30 ± 1.06	3.28 ± 1.10	2.60 ± 1.12	5.88	0.014*	Moderate vs. low intake (p < 0.05)	-0.39**	0.43**	-0.28*
Carrots (vitamin A)	2.20 ± 1.05	3.32 ± 1.08	2.50 ± 1.09	6.02	0.011*	High vs. low intake (p < 0.05)	-0.41**	0.46**	-0.31*
Eggs (vitamin D)	2.35 ± 1.10	3.15 ± 1.12	2.70 ± 1.14	4.55	0.026*	Moderate vs. low intake (p < 0.05)	-0.37**	0.39**	-0.23*
Milk & yogurt (vitamin D)	2.18 ± 1.07	3.45 ± 1.10	2.45 ± 1.12	7.50	0.007**	High vs. low intake (p < 0.01)	-0.47**	0.54**	-0.34**
Butter (vitamin D, E)	2.30 ± 1.09	3.20 ± 1.08	2.60 ± 1.10	5.12	0.019*	Moderate vs. low intake (p < 0.05)	-0.38**	0.40**	-0.27*
Avocado (vitamin E)	2.12 ± 1.05	3.55 ± 1.09	2.35 ± 1.08	8.80	0.004**	High vs. low intake (p < 0.01)	-0.50**	0.57**	-0.36**
Peanuts (vitamin E)	2.20 ± 1.08	3.38 ± 1.12	2.48 ± 1.11	6.35	0.010*	High vs. low intake (p < 0.01)	-0.42**	0.48**	-0.33**
Olives (vitamin E)	2.15 ± 1.06	3.50 ± 1.08	2.40 ± 1.09	7.90	0.005**	High vs. low intake (p < 0.01)	-0.49**	0.56**	-0.35**

Data in Table 8 reveal a significant negative correlation between nutrient-rich food intake and aggression levels, indicating that individuals who consume higher amounts of omega-3 fatty acids (fish, poultry, tofu, and beans), magnesium (nuts and seeds), and vitamins A, D, and E exhibit lower aggression scores. Specifically, vitamin E-rich foods (e.g., avocados, sunflower seeds) show the strongest protective effect (r = -0.50, p < 0.01), followed by vitamin D sources (r = -0.45, p < 0.01), suggesting their crucial role in mood regulation and impulse control. Omega-3 fatty acids (r = -0.42, p < 0.01) further contribute to emotional stability by reducing neuroinflammation, while vitamin A sources (r = -0.33, p < 0.05) also play a role in mitigating stress responses.

**Table 8:** Statistical analysis of correlation between food intake and aggression levels (n=219)

Food Category	Nutrient	Mean Intake (per week)	Correlation with Aggression (r value)	p-value
Omega-3 FA (fish, poultry, tofu, beans)	Omega-3 fatty acids	3.2 servings	-0.42	< 0.01
Nuts & seeds (almonds, walnuts, chia, flaxseeds)	Magnesium, antioxidants	4.1 servings	-0.39	< 0.01
Vitamin A sources (carrots, sweet potatoes, spinach)	Vitamin A	3.8 servings	-0.33	< 0.05
Vitamin D sources (milk, yogurt, butter, eggs)	Vitamin D	5.6 servings	-0.45	< 0.01
Vitamin E sources (avocados, olives, sunflower seeds)	Vitamin E	3.5 servings	-0.50	< 0.01

In Table 9, independent t-test results indicate that red meat consumers have significantly higher aggression scores (28.6 ± 5.2) compared to non-consumers (22.3 ± 4.8), with p < 0.01, suggesting a strong statistical difference. The effect size (Cohen’s d = 0.72) indicates a moderate-to-high impact, reinforcing that frequent red meat consumption is associated with increased aggression levels.

**Table 9:** Difference in aggression scores between red meat consumers and non-consumers (n = 219)

Group	(N=219)	Mean Aggression Score (± SD)	t-value	p-value	Effect Size (Cohen’s d)
Red meat consumers	115	28.6 ± 5.2	6.74	< 0.01	0.72 (moderate-high)
Non-red meat consumers	104	22.3 ± 4.8	NA	NA	NA

Table 10 highlights the impact of diet on behavioral traits, including aggression, temperament, and suspicion. Statistical analyses (ANOVA, correlation, and Tukey’s HSD) show that high intake of magnesium (r = -0.48, p < 0.01), vitamin E (r = -0.50, p < 0.01), and antioxidants (r = -0.46, p < 0.01) significantly reduces aggression by improving neurotransmitter function and lowering oxidative stress. Nutrient-rich foods like milk, yogurt, eggs, and oranges correlate with emotional stability (r = 0.38 to 0.57, p < 0.01), while unbalanced diets high in red meat and poultry contribute to mood fluctuations. Suspicion and resentment negatively correlate with high-nutrient intake (r = -0.22 to -0.36, p < 0.05), with ANOVA results (F = 4.55 to 8.80, p < 0.05) confirming these effects. While fish offers moderate benefits, it does not fully counteract the negative impact of excessive red meat. Overall, a balanced diet enhances emotional well-being, reduces aggression, and fosters social trust, underscoring the crucial role of nutrition in mental and behavioral health.

**Table 10:** Comparative analysis of nutrient-dense vs. red meat based diets on behavioral traits

Behavioral Trait	Healthy Intake (Magnesium, Vitamin E, Antioxidants, Dairy, Fruits, Vegetables)	Red Meat, Fish, and Poultry Intake
Aggression & violence	Lower aggression levels (r = -0.48 to -0.50, p < 0.01), improved neurotransmitter regulation, reduced impulsivity, and irritability.	Higher aggression levels due to increased oxidative stress and inflammatory responses, with minimal mood-stabilizing benefits.
Temperament & emotional response	Positive correlation with emotional stability (r = 0.38 to 0.57, p < 0.01), balanced temperament, and improved serotonin production.	Less consistent mood stability: while fish intake provides omega-3 benefits, excessive red meat may contribute to stress and anxiety.
Suspicion & resentment	Reduced paranoia and hostility (r = -0.22 to -0.36, p < 0.05), better trust levels, and lower anxiety.	Higher suspicion and social withdrawal tendencies, especially with excessive red meat intake. Fish intake may have a neutral to slightly positive impact.
Statistical significance	ANOVA (F = 4.55 to 8.80, p < 0.05), Post-hoc Tukey’s HSD confirms positive effects on mood and behavior.	Less statistically significant improvements compared to nutrient-dense foods, except for fish, which provides moderate benefits.

## Discussion

The study comprehensively analyzed the impact of dietary intake on aggression levels among Indian males aged 18 and above, revealing significant differences in behavioral outcomes based on food consumption patterns. Findings indicate that individuals consuming red meat reported the highest mean aggression score (28.6 ± 5.2), significantly higher than those who did not consume red meat (22.3 ± 4.8). This difference was confirmed using an independent t-test (t = 6.74, p < 0.01), demonstrating a statistically significant variation in aggression levels between red meat consumers and non-consumers. Cohen’s d value (0.72) suggests a moderate-to-high effect size, reinforcing the impact of red meat consumption on aggressive tendencies.

Current evidence suggests that lower omega-3 levels are linked to higher aggression. While research on the impact of vitamin D and zinc on aggressive behavior is more limited, there is preliminary evidence showing a negative association between these nutrients and aggression in both healthy individuals and psychiatric populations. The connection between magnesium and aggression varies depending on the method of assessment. Experimental trials indicate that nutritional interventions, such as omega-3 supplementation, could be an effective treatment, with benefits potentially extending beyond the intervention period [15]. Further analysis established strong associations between specific food groups and aggression levels. Red meat consumption exhibited a positive correlation with aggression (r = 0.48, p < 0.01), indicating that individuals who frequently consume red meat are more likely to display higher aggression scores. Conversely, the intake of omega-3 fatty acids (found in fish, nuts, and seeds) and vitamins A, D, and E (found in dairy, eggs, and leafy greens) demonstrated a negative correlation with aggression, ranging from r = -0.38 to r = -0.52 (p < 0.01). These findings suggest that nutrient-rich foods play a protective role in mitigating aggressive tendencies and promoting emotional stability.

The ANOVA results (F = 8.62, p < 0.01) further support the hypothesis that dietary habits significantly influence aggression levels. Post-hoc analysis using Tukey’s HSD test identified red meat consumers as the group with the highest aggression scores, followed by those consuming moderate amounts of poultry and fish, while individuals adhering to a nutrient-rich diet (high in omega-3, antioxidants, and vitamins) exhibited the lowest aggression levels. A significant amount of research indicates that diets rich in animal products may be linked to worse physical health [23,24]. Additionally, some evidence suggests that consuming animal-based foods could be connected to mental health problems like depression, potentially due to factors such as metabolic stress, obesity, and inflammation associated with their high fat content [25]. Comparative analysis confirms that individuals with higher fish and poultry consumption, rich in lean protein and omega-3 fatty acids, had significantly lower aggression scores compared to frequent red meat consumers. These results highlight the importance of dietary choices in shaping emotional and behavioral responses, emphasizing the need for greater awareness regarding the psychological effects of nutrition.

The statistical results reinforce the hypothesis that dietary choices play a critical role in shaping behavioral and psychological health. The higher aggression scores among red meat consumers suggest that excessive intake of saturated fats and iron-rich foods may contribute to neuroinflammation, oxidative stress, and hormonal imbalances, which in turn influence mood and aggression. Research indicates that red meat consumption leads to increased production of pro-inflammatory cytokines, which can impair neurotransmitter function, particularly serotonin and dopamine, both of which are crucial for emotional regulation [26]. Additionally, higher levels of arachidonic acid (found in red meat) are linked to increased stress responses and heightened aggression. These findings align with previous studies demonstrating that diets rich in processed and high-fat meats correlate with impulsivity, irritability, and aggressive behavior [27].

In contrast, the negative correlation between omega-3 fatty acids, vitamins A, D, and E, and aggression levels suggests that these nutrients play a protective role in mental health. Omega-3 fatty acids, primarily found in fish, nuts, and seeds, are essential for reducing inflammation and improving neurotransmitter function, particularly in regulating serotonin and dopamine activity [28]. This contributes to better mood stability, reduced impulsivity, and lower aggression levels [29]. The findings from Table 8 and Table 9 confirm that individuals with a diet rich in omega-3 and antioxidants show lower aggression scores and improved emotional regulation compared to those with high red meat intake.

However, the ANOVA and Tukey’s HSD test results highlight significant behavioral differences among dietary groups, indicating that the frequency and type of food intake are crucial factors influencing aggression levels. Individuals following a diet high in vitamin D (milk, eggs), vitamin E (nuts, seeds), and vitamin C (citrus fruits, vegetables) exhibit enhanced mood stability and reduced aggression, likely due to their role in modulating cortisol levels, reducing oxidative stress, and supporting serotonin synthesis [30]. This suggests that adopting a diet focused on anti-inflammatory and nutrient-dense foods could serve as an effective strategy for reducing aggressive behaviors and promoting psychological well-being.

Multiple studies indicate that lower levels of omega-3 fatty acids, vitamin D, and zinc are associated with increased aggression, a trend observed in both healthy individuals and those with psychiatric conditions [29,30]. Additionally, lower magnesium intake has been associated with increased aggression, though results are inconsistent when different measures of magnesium are used. The most substantial evidence comes from omega-3 fatty acid studies, which show that supplementation for at least six weeks can reduce aggressive behavior, with early indications that its effects persist after treatment ends.

The overall findings of the study emphasize that diet plays a pivotal role in regulating aggression and emotional responses. The significant correlations between food intake and aggression levels provide strong evidence that reducing red meat consumption while increasing the intake of omega-3-rich foods, antioxidants, and vitamins can help mitigate aggressive tendencies and promote emotional resilience. These insights highlight the importance of nutritional interventions in behavioral management and mental health strategies. This study suggests promoting healthier diets to reduce aggression and improve psychological well-being. It emphasizes the benefits of omega-3 fatty acids (found in fish, nuts, and seeds) for lowering aggression while recommending reduced intake of red meat, particularly processed ones, in favor of healthier protein sources like poultry, fish, tofu, and legumes. Antioxidant-rich foods, such as those high in vitamins A, D, and E, should also be incorporated to support emotional stability. Public health campaigns should raise awareness about the psychological impacts of food choices, and nutrition education should be included in school curricula. Further research is needed on the long-term effects of diet on aggression, mood disorders, and emotional regulation.

Limitations

The present study has some limitations also. Although a random sampling approach was used, participants were recruited from an opt-in online research panel, which may not be fully representative of the general male population in India. This could limit the generalization of the findings. While demographic data were collected, the study did not fully account for potential confounding factors such as physical activity, mental health status, socioeconomic status, or substance use, which could influence aggression levels. Both dietary intake and aggression levels were assessed using self-reported questionnaires (FFQ and BPAQ), which are subject to recall bias, social desirability bias, and misreporting, potentially affecting data accuracy. The study focused solely on males aged 18 and above, which limits the applicability of the findings to females or younger populations, potentially reducing the external validity.

## Conclusions

This study highlights the impact of diet on aggression levels among Indian male participants aged 18 and above. It found that red meat consumption is linked to higher aggression. On the other hand, diets high in omega-3 fatty acids, vitamins A, D, and E, and antioxidants are linked to reduced aggression. Statistical analyses confirm that diets high in anti-inflammatory nutrients improve emotional regulation. The study emphasizes the importance of diet in mental health and suggests that reducing red meat and increasing nutrient-rich foods can enhance mood stability and well-being. These findings support the need for nutrition-based public health strategies to promote better behavioral outcomes. Moving forward, greater awareness and education on the link between diet and psychological health can help individuals make informed dietary choices, ultimately fostering a more balanced and emotionally stable society.

## Appendices

Appendix A

Questionnaire to assess the correlation between Diet and Aggression

1)        Age (years)

1.        16-20

2.        21-24

3.        25-29

4.        30-34

5.        35 and above

2)       Education

1.        Illiterate

2.        Primary school

3.        High school

4.        Intermediate

5.        Graduation

6.        post-graduation

7.        PhD

3)       Marital status

1.        single

2.       Married

3.       Separated/divorced

4.        Prefer not to say

4)       Occupation

1.        Academic

2.        Professional

3.        Corporate

4.        Business

5.        Student

6.        Unemployed

5)       Please indicate how often you consume each of the following foods: 

Red meat

1.        Never

2.       Rarely (1-2 times/month)

3.       Occasionally (1-2 times/week)

4.       Often (3-4 times/week)

5.        Very often (5-6 times/week)

6.        Daily (7 times/week)

Poultry

1.        Never

2.        Rarely (1-2 times/month)

3.        Occasionally (1-2 times/week)

4.        Often (3-4 times/week)

5.        Very often (5-6 times/week)

6.        Daily (7 times/week)

Fish

1.        Never

2.        Rarely (1-2 times/month)

3.        Occasionally (1-2 times/week)

4.        Often (3-4 times/week)

5.        Very often (5-6 times/week)

6.        Daily (7 times/week)

Beans and Lentils

1.        Never

2.        Rarely (1-2 times/month)

3.        Occasionally (1-2 times/week)

4.        Often (3-4 times/week)

5.        Very often (5-6 times/week)

6.        Daily (7 times/week)

Spina

1.        Never

2.        Rarely (1-2 times/month)

3.        Occasionally (1-2 times/week)

4.        Often (3-4 times/week)

5.        Very often (5-6 times/week)

6.        Daily (7 times/week)

Tofu/ Paneer

1.        Never

2.        Rarely (1-2 times/month)

3.        Occasionally (1-2 times/week)

4.        Often (3-4 times/week)

5.        Very often (5-6 times/week)

6.        Daily (7 times/week)

Dry Fruits

1.        Never

2.        Rarely (1-2 times/month)

3.        Occasionally (1-2 times/week)

4.        Often (3-4 times/week)

5.        Very often (5-6 times/week)

6.        Daily (7 times/week)

Chia seeds / Flaxseeds

1.        Never

2.        Rarely (1-2 times/month)

3.        Occasionally (1-2 times/week)

4.        Often (3-4 times/week)

5.        Very often (5-6 times/week)

6.        Daily (7 times/week)

Magnesium-Rich Foods  ( black beans / dark chocolates)

1.        Never

2.        Rarely (1-2 times/month)

3.        Occasionally (1-2 times/week)

4.        Often (3-4 times/week)

5.        Very often (5-6 times/week)

6.        Daily (7 times/week)

Oranges (Vitamin C)

1.        Never

2.        Rarely (1-2 times/month)

3.        Occasionally (1-2 times/week)

4.        Often (3-4 times/week)

5.        Very often (5-6 times/week)

6.        Daily (7 times/week)

Tomatoes ( Vitamin C, A)

1.        Never

2.        Rarely (1-2 times/month)

3.        Occasionally (1-2 times/week)

4.        Often (3-4 times/week)

5.        Very often (5-6 times/week)

6.        Daily (7 times/week)

Carrots (Vitamin A)

1.        Never

2.        Rarely (1-2 times/month)

3.        Occasionally (1-2 times/week)

4.        Often (3-4 times/week)

5.        Very often (5-6 times/week)

6.        Daily (7 times/week)

Eggs (Vitamin D)

1.        Never

2.        Rarely (1-2 times/month)

3.        Occasionally (1-2 times/week)

4.        Often (3-4 times/week)

5.        Very often (5-6 times/week)

6.        Daily (7 times/week)

Milk and Yogurt (Vitamin D)

1.        Never

2.        Rarely (1-2 times/month)

3.        Occasionally (1-2 times/week)

4.        Often (3-4 times/week)

5.        Very often (5-6 times/week)

6.        Daily (7 times/week)

Butter (Vitamin D, E)

1.        Never

2.        Rarely (1-2 times/month)

3.        Occasionally (1-2 times/week)

4.        Often (3-4 times/week)

5.        Very often (5-6 times/week)

6.        Daily (7 times/week)

Avocado (Vitamin E)

1.        Never

2.        Rarely (1-2 times/month)

3.        Occasionally (1-2 times/week)

4.        Often (3-4 times/week)

5.        Very often (5-6 times/week)

6.        Daily (7 times/week)

Peanuts (Vitamin E)

1.        Never

2.        Rarely (1-2 times/month)

3.        Occasionally (1-2 times/week)

4.        Often (3-4 times/week)

5.        Very often (5-6 times/week)

6.        Daily (7 times/week)

Olives (Vitamin E)

1.        Never

2.        Rarely (1-2 times/month)

3.        Occasionally (1-2 times/week)

4.        Often (3-4 times/week)

5.        Very often (5-6 times/week)

6.        Daily (7 times/week)

## References

[1] Gibson EL (2006). Emotional influences on food choice: sensory, physiological and psychological pathways. Physiol Behav.

[2] Spencer RL, Deak T (2017). A users guide to HPA axis research. Physiol Behav.

[3] Heidari M, Khodadadi Jokar Y, Madani S, Shahi S, Shahi MS, Goli M (2023). Influence of food type on human psychological-behavioral responses and crime reduction. Nutrients.

[4] American Psychiatric Association (1987). Diagnostic and Statistical Manual of Mental Disorders, Third Edition. Diagnostic and Statistical Manual of Mental Disorders, Third Edition.

[5] van den Akker N, Kroezen M, Wieland J, Pasma A, Wolkorte R (2021). Behavioural, psychiatric and psychosocial factors associated with aggressive behaviour in adults with intellectual disabilities: a systematic review and narrative analysis. J Appl Res Intellect Disabil.

[6] Crotty G, Doody O, Lyons R (2014). Aggressive behaviour and its prevalence within five typologies. J Intellect Disabil.

[7] da Silva MC, Canário AV, Hubbard PC, Gonçalves DM (2021). Physiology, endocrinology and chemical communication in aggressive behaviour of fishes. J Fish Biol.

[8] Mazur A, Booth A (1998). Testosterone and dominance in men. Behav Brain Sci.

[9] Archer J (2006). Testosterone and human aggression: an evaluation of the challenge hypothesis. Neurosci Biobehav Rev.

[10] van Honk J, Schutter DJ, Hermans EJ, Putman P, Tuiten A, Koppeschaar H (2004). Testosterone shifts the balance between sensitivity for punishment and reward in healthy young women. Psychoneuroendocrinology.

[11] Higley JD, Mehlman PT, Poland RE (1996). CSF testosterone and 5-HIAA correlate with different types of aggressive behaviors. Biol Psychiatry.

[12] Kaye WH, Frank GK, Bailer UF (2005). Serotonin alterations in anorexia and bulimia nervosa: new insights from imaging studies. Physiol Behav.

[13] Bailer UF, Frank GK, Henry SE (2005). Altered brain serotonin 5-HT1A receptor binding after recovery from anorexia nervosa measured by positron emission tomography and [carbonyl11C]WAY-100635. Arch Gen Psychiatry.

[14] Delvenne V, Lotstra F, Goldman S (1995). Brain hypometabolism of glucose in anorexia nervosa: a PET scan study. Biol Psychiatry.

[15] Choy O (2023). Nutritional factors associated with aggression. Front Psychiatry.

[16] Werbach M (1992). Nutritional influences on aggressive behavior. J Orthomol Med.

[17] Weightman M, Kini R, Parker R, Das M (2020). Pharmacological approaches to managing violence and aggression in prison populations: clinical and ethical issues. Drugs.

[18] van Schalkwyk GI, Beyer C, Johnson J, Deal M, Bloch MH (2018). Antipsychotics for aggression in adults: a meta-analysis. Prog Neuropsychopharmacol Biol Psychiatry.

[19] Zaalberg A, Wielders J, Bulten E, van der Staak C, Wouters A, Nijman H (2016). Relationships of diet-related blood parameters and blood lead levels with psychopathology and aggression in forensic psychiatric inpatients. Crim Behav Ment Health.

[20] Mommaerts K, Lopez NV, Camplain C, Keene C, Hale AM, Camplain R (2023). Nutrition availability for those incarcerated in jail: Implications for mental health. Int J Prison Health.

[21] Buss AH, Perry M (1992). The Aggression Questionnaire. J Personal Soc Psychol.

[22] Gravetter FJ, Wallnau LB (2021). Statistics for the Behavioral Sciences: 10th Edition. http://ndl.ethernet.edu.et/bitstream/123456789/29095/1/Frederick%20J%20Gravetter_2017.pdf.

[23] Cross AJ, Leitzmann MF, Gail MH, Hollenbeck AR, Schatzkin A, Sinha R (2007). A prospective study of red and processed meat intake in relation to cancer risk. PLoS Med.

[24] Larsson SC, Wolk A (2006). Meat consumption and risk of colorectal cancer: a meta-analysis of prospective studies. Int J Cancer.

[25] Nucci D, Fatigoni C, Amerio A, Odone A, Gianfredi V (2020). Red and processed meat consumption and risk of depression: a systematic review and meta-analysis. Int J Environ Res Public Health.

[26] Zhang Y, Yang Y, Xie MS, Ding X, Li H, Liu ZC, Peng SF (2017). Is meat consumption associated with depression? A meta-analysis of observational studies. BMC Psychiatry.

[27] Montgomery DC (2020). Design and Analysis of Experiments.

[28] Hair JF, Black WC, Babin BJ, Anderson RE (2019). Multivariate Data Analysis, Eighth Edition. https://eli.johogo.com/Class/CCU/SEM/_Multivariate%20Data%20Analysis_Hair.pdf.

[29] Cohen J, Cohen P, West SG, Aiken LS (2013). Applied Multiple Regression/Correlation Analysis for the Behavioral Sciences.

[30] Dawson LG (2021). Excel Manual for Moore and McCabe’s Introduction to the Practice of Statistics: Fifth Edition. W. H. Freeman.

